# Heat Adaptation in Military Personnel: Mitigating Risk, Maximizing Performance

**DOI:** 10.3389/fphys.2019.01485

**Published:** 2019-12-17

**Authors:** Iain T. Parsons, Michael J. Stacey, David R. Woods

**Affiliations:** ^1^Academic Department of Military Medicine, Research and Clinical Innovation, Royal Centre for Defence Medicine, Birmingham, United Kingdom; ^2^School of Cardiovascular Medicine & Sciences, Faculty of Life Sciences & Medicine, King’s College London, London, United Kingdom; ^3^Department of Diabetes and Endocrinology, Imperial College Healthcare NHS Trust, London, United Kingdom; ^4^Department of Sport and Exercise Endocrinology, Carnegie Research Institute, Leeds Beckett University, Leeds, United Kingdom

**Keywords:** heat acclimation, heat acclimatization, heat adaptation, heat stroke, heat syncope, heat illness, heat stress

## Abstract

The study of heat adaptation in military personnel offers generalizable insights into a variety of sporting, recreational and occupational populations. Conversely, certain characteristics of military employment have few parallels in civilian life, such as the imperative to achieve mission objectives during deployed operations, the opportunity to undergo training and selection for elite units or the requirement to fulfill essential duties under prolonged thermal stress. In such settings, achieving peak individual performance can be critical to organizational success. Short-notice deployment to a hot operational or training environment, exposure to high intensity exercise and undertaking ceremonial duties during extreme weather may challenge the ability to protect personnel from excessive thermal strain, especially where heat adaptation is incomplete. Graded and progressive acclimatization can reduce morbidity substantially and impact on mortality rates, yet individual variation in adaptation has the potential to undermine empirical approaches. Incapacity under heat stress can present the military with medical, occupational and logistic challenges requiring dynamic risk stratification during initial and subsequent heat stress. Using data from large studies of military personnel observing traditional and more contemporary acclimatization practices, this review article (1) characterizes the physical challenges that military training and deployed operations present (2) considers how heat adaptation has been used to augment military performance under thermal stress and (3) identifies potential solutions to optimize the risk-performance paradigm, including those with broader relevance to other populations exposed to heat stress.

## Introduction

Heat stress, through occupational exposure to strenuous physical exercise and/or environmental extremes of heat and humidity, presents a perennial challenge (Hancock et al., 2007) to military personnel. The consequences of incomplete or inadequate heat adaptation may be fatal; either directly, as with heat stroke, or through impaired physiological functioning and increased susceptibility to military hazards, including combat. Augmenting physical and mental performance through heat adaptation may be pivotal to military operational success (Nindl et al., 2013).

Over the last two decades, many Western militaries have conducted land-based campaigns in climatically-severe regions (World and Booth, 2008; Cox et al., 2016a). These operations frequently evolved into mature missions allowing for targeted physical training prior to deployment and large-scale graded acclimatization practices upon arrival to operational theaters. In more recent history, several militaries have contributed to internationally-sponsored security projects including the protection of internally displaced civilian populations (Bailey et al., 2019) and the containment of infectious diseases outbreaks, such as Ebola (Dickson et al., 2018). Achieving peak occupational performance and developing and maintaining resilience in the face of thermal threat has been a critical endpoint in all such circumstances.

Routinely, the ‘special population’ fulfilling these roles is drawn from the general population of its parent nation and allied entities. Historically, the study of military personnel has provided insights into acclimatization mechanisms and strategies that have been generalizable to other occupational groups and the wider civilian population (Conn, 1963). The concept of relative climatic adaptation has been attributed to the renowned British Naval officer and clinician-scientist, Sir James Lind (Lind, 1771; Périard et al., 2015). As research findings on heat adaptation in this special population may be applied in other groups, so those learned elsewhere may have potential to prove militarily advantageous.

Illustrated by research data from uniformed personnel observing traditional and more contemporary acclimatization practices – and with particular reference to recent United Kingdom (UK) military experience – this article (1) characterizes the physical challenges that modern training and operations present (2) considers how heat adaptation has been used or may be applied to protect and augment the health and performance of military personnel and (3) identifies potential solutions to optimize the risk-performance paradigm that operates both during and after heat acclimatization.

## Methods

No articles were dismissed in this review but, where appropriate, comment has been made on the limitations of the study presented, i.e., limited subject numbers and confounding methodological issues. Historically, based journals have been included where appropriate to indicate the limitations in current research. Use of the terms ‘military,’ ‘armed forces,’ ‘heat acclimation,’ ‘heat acclimatis(z)ation,’ heat adaptation and ‘heat illness’ were employed. Search engines included PubMed, Web of Knowledge and directly from the appropriate academic journals. Studies previously reported from our group’s work with the United Kingdom military are used to illustrate certain concepts, including novel unpublished data.

## The Thermal Challenge of Modern Military Operations, Training, and Ceremonial Duties

In a volatile and increasingly complex world, governments of globally-facing nations foster contingency for a wide variety of overseas commitments (Ministry of Defence, 2018). These may be conducted through changing weather and elevation, over a range of timescales and set across challenging geographies: from short notice deployments to more enduring overseas operations (Edholm, 1969; Shapiro et al., 1981). Performance requirements are equally diverse arising from the scope of potential missions confronting the military and associated bodies, such as host nation civilian agencies and non-governmental aid organizations. Dedicated military taskings may include training and mentoring to allied forces; counter-terrorism/insurgency work; and full-scale war, including ‘peer-to-peer’ conflict conducted at large-scale, high tempo and over substantial physical areas. Other activities in which the military may contribute, or play a complementary role, include medical and engineering support to humanitarian relief efforts; law enforcement; firefighting responses; and management of other natural or man-made disasters and hazards (Ministry of Defence, 2018).

Operational capability may be impaired when unacclimatized personnel are required to travel at short-notice to a severe climate or to over-ride intrinsic or extrinsic thermal safeguards in the face of physical or tactical threats. For example, operational pressures may on occasion limit time or opportunity for full acclimatization, in favor of getting ‘boots on the ground’ with a view to achieving immediate military effects. From a physiological standpoint, however, inadequate or sub-optimal heat adaptation may have an array of consequences, the most feared of which is heat stroke and its associated morbidity and mortality (Abriat et al., 2014). Heat stroke has been defined as central nervous system (CNS) dysfunction, multiorgan failure and extreme hyperthermia, with core body temperature (Tc) usually > 40.5°C (Epstein and Yanovich, 2019). It arises from failure to dissipate excessive body heat and is presently considered more preventable than treatable, being associated with a high mortality rate where treatment is delayed (Leon and Bouchama, 2015). Heat stroke and lesser forms of incapacity may be defined occupationally as ‘heat illness’: an ‘all embracing term including those individuals who become incapacitated through exhaustion or syncope as a result of a rise in core body temperature’(Ministry of Defence, 2017). The occurrence of heat illness in military personnel falls with heat acclimation/acclimatization (Bean and Eichna, 1943; Edholm, 1969). This is particularly-well evidenced for minor episodes of heat illness characterized by heat syncope, or syncope upon cessation of exercise (post-exertional syncope), with prompt recovery of consciousness on prostration (Bean and Eichna, 1943). It is less clear whether risk of more severe illness, such as heat stroke, is substantially ameliorated in the heat adapted phenotype (Lim and Mackinnon, 2006). An increased hospitalization risk for heat illness (as a surrogate for severity) has been reported in unselected military personnel considered acclimatized by attending physicians (Stacey et al., 2015). Hospitalization (Carter et al., 2005), and heat stroke deaths (Malamud et al., 1946) are less prevalent in military recruit populations originating from hotter regions, as opposed to cooler. However, it is unclear whether this is secondary to long-term physiological adaptation and/or modification of exertional behaviors and practices. In military recruit populations training in hot regions 77% of cases of heat illness (all forms) and 75% of heat stroke deaths have been observed to occur in the first 8 weeks of training, though the remainder of cases after this point are not insignificant proportions, considering the expected time course for acclimatization (Borden et al., 1945).

When an episode of heat illness occurs during, or soon after, strenuous physical exertion, it may be termed Exertional Heat Illness (EHI). This has been the predominant form of heat illness to affect British Army personnel in recent history, with EHI representing 96% of military cases reported by military doctors between 2009 and 2013 (Stacey et al., 2015) and continuing to make up the majority of referrals for specialist assessment. Most United Kingdom cases are incapacitated during training in temperate climates (Stacey et al., 2016). However, during the first 6 months of operational deployment to the extreme-dry heat of Iraq in 2003, 849 heat-implicated casualties were reported among United Kingdom military personnel, requiring 766 hospital admissions and 161 aeromedical evacuations to the United Kingdom (Bricknell, 2003). Three hundred of these cases presented to a dedicated Heat Illness Unit in July alone (incidence 50 per 1,000 deployed personnel) (Bolton et al., 2006) 90% had been affected within the first 10 days of arrival into theater and only a minority (14%) had been undertaking heavy work at the time of incapacity. This experience was reflected in Schickele’s historic 1947 analysis of fatal heat stroke among military recruits in the continental United States, in which over 80% of 157 cases confirmed at post-mortem suffered heat stroke in relation to ‘average activity’ (drill, guard duty, relatively short marches) versus only 14% affected during heavy exercise (Schickele, 1947). Nearly all deaths occurred above a critical threshold of elevated heat and/or humidity, though some cases occurred with heavy physical exercise below the ‘heat death line.’ At the multinational field hospital in Camp Bastion, Afghanistan, heat illness was also a leading cause of admission, ranking second only to infectious diseases among all diagnoses made in internal medicine (2011 to 2013, summer and non-summer presentations) (Cox et al., 2016a). In the experience of the authors, the case load in Afghanistan represented a mixture of exertional, non-exertional, and intermediate heat illness (i.e., incapacitation where only light levels of physical activity were implicated in pathogenesis). Thus incapacity sustained with exposure to heat stress may manifest with excessive external (climatic, both macro and microclimate) and internal (metabolic) load, with each type of stressor capable of causing incapacity at lower ‘doses’ when operating synergistically. Heat stress also impacts on other duties, particular to the military, such as precipitating syncope or heat exhaustion during drill (Smalley et al., 2003; Wallace et al., 2006; Budd, 2008) often during large state ceremonial parades (Parsons et al., 2015). The effect of heat stress on reducing orthostatic tolerance has been well-demonstrated (Crandall et al., 2010a; Crandall and Wilson, 2015; Schlader et al., 2016a, b).

## Heat Adaptation: Health Protection and Augmented Performance in Military Personnel

The thermal challenge of military service raises questions over the optimal ways for parent organizations to exercise their duty of care and facilitate mission objectives. Heat adaptation is a key component of managing the overarching risk presented and, in the military as elsewhere, serves two key purposes: (1) the prevention of heat-related illnesses and (2) the improvement of physical performance and mental functioning. Crucially for military tasks, this may include improved decision-making under heat stress (Cheung et al., 2016).

Acquisition of the heat-adapted state occurs through repeated or continuous exposure to heat stress and accompanying elevation in Tc and may be achieved with artificial exposure to heat, by residence in a natural hot climate, or with physical training sufficient to raise Tc in less severe conditions (Leon and Bouchama, 2015). Phenotypic changes that are associated with repeated heat stress include altered sweating and skin blood flow responses, decreased metabolic rate, plasma volume (PV) expansion and improved cardiovascular stability (Nielsen, 1998; Shapiro et al., 1998; Patterson et al., 2004). In the hot environment, acquisition of these adaptations associates with improved thermal comfort (Sato et al., 1990), lower physiological strain (Taylor, 2014) and restored performance capacity for equivalent bouts of exercise conducted in less severe conditions. In some studies, performance is also enhanced on return to the thermoneutral environment, by way of increased maximal aerobic capacity (Sawka et al., 1985; Lorenzo et al., 2010). A core physiological change in heat adaptation, as described by Conn and Johnston (1944), is increased sweat rate and decreased concentration of sweat sodium and chloride (Leon and Bouchama, 2015) due to changes in the eccrine glands (Sato et al., 1990). Sweating, through changes in the CNS, occurs earlier and at a lower core temperature (Taylor, 2014).

Bass and Henschel defined acclimatization as ‘the dramatic improvement in the ability to work in the heat which occurs within 4 to 7 days of first exposure’ (Bass and Henschel, 1956). Over this period, rapid, demonstrably effective (Garrett et al., 2009, 2012) but ultimately incomplete (Pandolf, 1998; Gibson et al., 2015) adaptation ensues with improved cardiovascular stability and heart rate decrease, in association with PV expansion (Armstrong and Maresh, 1991). Horvath and Shelley described attaining a stable state of improved physical and mental functioning with fuller acclimatization (Horvath and Shelley, 1946) encompassing the features of longer term heat adaptation such as behavior changes resulting in reduced heat stress, e.g., reduced work and greater use of shelter (Périard et al., 2015), and improved thermal sensation (Chalmers et al., 2014). Acclimation status has been categorized by the number of exposures to heat stress (Chalmers et al., 2014; Tyler et al., 2016) as short-term (up to seven exposures), medium- term (eight to 14 exposures), and long-term (15 or more exposures).

**FIGURE 1:**
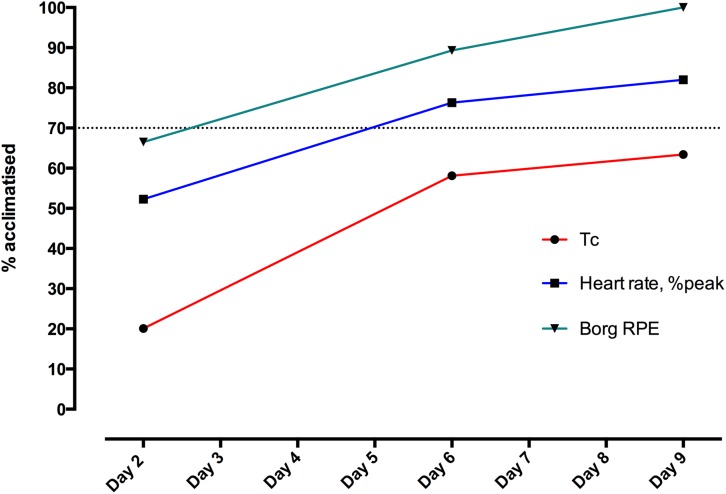
Heat adaptation in 21 volunteers, displayed as progress toward full (100%) acclimatization status on Day 23 in Cyprus, relative to pre-deployment baseline in United Kingdom (0% acclimatized). Volunteers performed the acclimatization protocol described in Table 1, with 60 min stepping at 50% VO_2_max in a climatic chamber controlled to WBGT 34°C, at United Kingdom baseline and on Days 2, 6, 9, and 23 following arrival in Cyprus (substituted for usual acclimatization exercise on these days). Acclimatization status (%) was calculated by parameter, in terms of the end-exercise value relative to that on Day-23. Tc, core body temperature; Borg RPE (Borg, 1973), Relative Perceived Exertion according to Borg’s scale (may need to reference: as below). Dotted line indicates 70% adaption.

Heat adaptation from exercise-heat stress is commonly induced through three mechanisms (Daanen et al., 2018): (1) constant work-rate exercise (Nadel et al., 1974; Nielsen et al., 1993, 1997) (2) self-paced exercise (Nelms and Turk, 1972) and (3) isothermic acclimation (Regan et al., 1996; Garrett et al., 2009). Additional novel methods that have been proposed include matching exercise intensity to the observed decrease in heart rate (Périard et al., 2015; Tyler et al., 2016) or constant-rate exercise to a fixed rating of perceived exertion (RPE) (Omassoli et al., 2019). For the purposes of simplicity and economy, military organizations have favored standardized exposures (Gibson et al., 2015), commonly at a constant work-rate (Daanen et al., 2018), with some approaches being more self-paced. In recent decades, Western military forces have regained significant experience in deploying large numbers of personnel to thermally-stressful environments for demanding combat and peace support operations. This has seen the introduction of empirical guidance to achieve safe and effective group-based acclimatization, drawing on earlier lessons learned in the wake of hot weather training and campaigns during World War 2 (Bean and Eichna, 1943; Schickele, 1947; Conn, 1963), and the propagation of general principles in support of this aim (Table 1). In this context, it has been stated that approximately 2 weeks of progressive heat exposure and physical work should expedite near-complete acclimatization, with the average service person expected to have achieved ∼50 and ∼80% of ultimate physiological adaptation, respectively, by the completion of the first and second weeks of appropriate training (STO/NATO, 2013) (Figure 1). An example of a validated acclimatization schedule for physically fit personnel to follow on arrival to a hot region is provided at Table 2. This is reproduced from United Kingdom Defense policy on prevention of climatic illness and injury (Ministry of Defence, 2017). The specific impact and potential implications of following this protocol are discussed below.

**TABLE 1:** Heat acclimatization strategies that can be considered before and after military deployment to a hot region (STO/NATO, 2013).

(1) Mimic the deployment climate.
(2) Ensure adequate heat stress by:
• Invoking profuse sweating.
• Using exercise and rest to modify the heat strain.
• Having 4 to 14 days of heat exposures.
• Maintaining the daily duration of at least 120 min.
(3) Start early (1 month before deployment).
• Performance benefits may take longer than physiological benefits.
• Be flexible with training.
• Build confidence.
• Pursue optimum physical fitness in the current climate.
(4) Methods.
• Pre-deployment: climate controlled room or hot weather.
• Integrate with training by adding additional acclimatization sessions; inserting.
• Acclimatization with training; alternating acclimatization days with training days, and no detraining.
• Mimic the deployment environment by working out in a warm room wearing sweats (*sic*) if you are in a cool/temperate environment.
(5) On arrival.
• Start slowly at reduced training intensity and duration and limit heat exposure.
• Increase heat and training volume (intensity and duration) as tolerance permits.
• Acclimatize in heat of day.
• Physical training should be conducted in coolest part of day.
• Use work/rest cycles or interval training.
• Be especially observant of salt needs for the first week of acclimatization.
• Sleeping in cool or air-conditioned rooms will not affect heat acclimatization status and will aid recovery from heat stress.

**TABLE 2:** A validated acclimatization schedule for physically fit personnel to follow on arrival to a hot region reproduced from United Kingdom Defense policy on prevention of climatic illness and injury (Ministry of Defence, 2017).

**Day**	**Dress**	**Target WBGT Index (°C WGBT)**	**Duration (min)**	**Activity**
1	No activity. rest, eat, drink, and sleep (for 24 h)			
2	T-shirt and shorts	26–30	1 × 50	Walk at 6 km/h (3.7 miles/h)
3	T-shirt and shorts	26–30	2 × 50	Walk at 6 km/h; rest for 15 min; resume walking.
4	T-shirt and shorts	26–30	100	Walk at 6 km/h
5	T-shirt, combat jacket, lightweight trousers, and body armor	26–30	2 × 50	Walk at 6 km/h for 50 min then remove body armor and jacket and rest for 15 min; resume walking.
6	T-shirt, combat jacket, lightweight trousers, and body armor	26–30	100	Walk at 6 km/h
7	T-shirt, combat jacket, lightweight trousers and body armor and webbing (10 kg)	26–30	2 × 50	Walk at 6 km/; remove webbing, rest for 15 min; resume walking.
8	T-shirt, combat jacket, lightweight trousers and body armor and webbing (10 kg)	26–30	100	Walk at 6 km/h
Personnel undertaking acclimatization should be allowed fluids as required				

Over the short term moderate intensity exercise appears to induce a more efficient physiological adaptation (Houmard et al., 1990) in terms of time spent exercising in the heat. But, for simple approaches utilizing constant work rate, it has been argued that the ‘forcing function’ of fixed environmental heat exposure and endogenous heat production is attenuated by the effects of progressive heat adaptation, in a process of habituation (Taylor, 2000, 2014). Such protocols may be convenient to apply and effective at restoring the ability to perform a standard bout of work in the new (hot) conditions, but may limit or slow progress toward maximum acclimatization. Furthermore, excessive thermal stress in the absence of close monitoring – as may be seen with large numbers of military participants working to a constant level or group pace, especially early in the course of adaptation – has potential to induce significant heat-related morbidity, with group-paced activities associating with hospitalization among United Kingdom heat illness casualties (Abriat et al., 2014). Yet whilst a self-paced heat acclimatization program would be seemingly protective in this regard, such practices are not an absolute safeguard, with soldiers exceeding pre-defined heart rate and temperature limits, particularly in competitive groups (Armstrong et al., 1986).

Isothermic or controlled hyperthermic acclimation commonly manipulates the endogenous thermal load under constant exogenous heat stress (Regan et al., 1996), but can also vary environmental conditions to reach and sustain a target Tc. These regimens associate with greater physiological adaptations in a shorter space of time, with incorporation of physical exercise (Hellon et al., 1956; Bain et al., 2013; Charlot et al., 2017) being more efficient than a passive strategy (Harrison, 1985; Périard et al., 2015; Racinais et al., 2015; Tyler et al., 2016). Often isothermic regimens utilize both forms of heat exposure (Daanen et al., 2018). Approaches of this kind could hold considerable appeal for military planners and commanders intent on taking personnel through a rapid transition to a more severe environment, as with deployment of high readiness troops into an emerging or escalating operational theater. This methodology is resource intensive however and may be unsuited to large scale implementation, i.e., in groups of military personnel above sub-unit size (20–30 pax). Whether it would always be operationally effective to strictly observe sub-maximal physiological thresholds is a matter of military debate, at least as it applies to routine training in the heat (Hunt et al., 2016). Attaining measures of heart rate, Tc or derived/composite parameters such as the Physiological Strain Index (Buller et al., 2018) could ensure safe initial induction of heat adaptation, whatever the method selected to achieve it.

## Practices and Pitfalls in Generating and Sustaining Heat Adaptation in Military Populations

Drawing parallels with civilian athletic groups, whom may also be rapidly required to perform in hot climates with minimal preparation (Périard et al., 2015), military personnel should maintain a high state of physical fitness. Military guidance developed within the North Atlantic Treaty Organization (NATO) (Figure 1) identifies an increased need for monitoring ‘the least fit soldier, who will have the most difficult time (to acclimatize) (STO/NATO, 2013). This assertion is supported by findings that: (1) physical fitness improves physiological responses to exercise in the heat (Armstrong and Maresh, 1991) (2) heat-related illnesses are reported at lower rates in trained personnel than (putatively less well-conditioned) recruits undergoing induction to the Armed Forces (Carter et al., 2005; Stacey et al., 2015) (3) aerobically well-trained participants may be ‘primed’ to heat adaptation with an indirect relationship existing between VO_2_max and the number of days required to acclimatize (Pandolf et al., 1977; Garrett et al., 2011). The NATO guidance also identifies a second high-risk group: ‘the most motivated soldiers, who may overdo their physical activity and be susceptible to becoming heat casualties’ (STO/NATO, 2013). Highly motivated personnel can be found throughout any military organization, but are often concentrated among the fittest elements, including the infantry, marines, and other ‘frontline’ occupations. These units are trained to engage in dismounted ground close combat (DGCC) and may be required to close with and kill enemy forces, among other physically-arduous functions. Reported heat stroke rates have been 7- to 11- times higher in the US Army and Marine Corps than in the Navy or Air Force and all forms of heat illness are significantly commoner in combat versus other occupational roles (Armed Forces Health Surveillance Bureau, 2019). Individuals in these higher risk groups may be required to deploy more quickly, risking inadequate time for acclimatization before action, plus exposure to a multitude of other factors that may delay or interrupt full heat adaptation. Whilst in most cases one single predisposing factor (Epstein, 1990; Epstein et al., 1999) is sufficient to reduce the ability of the individual to tolerate heat during physical activity – or even with passive exposure – aggregating factors can further increase risk for heat illness (Gardner et al., 1996). Motivation to accomplish a mission, whilst disregarding early signs and symptoms for heat-related disability, is a contributing factor that can significantly exacerbate this situation (Shibolet et al., 1976; Epstein et al., 1999).

In advance of deployment to a hot environment, United Kingdom military guidance directs commanders, physical training staff and individual service personnel to build or improve aerobic fitness over 3 to 4 weeks, aiming to ‘increase and maintain heart rate above 65% of maximum heart rate for periods of 40 min initially, then extending to an hour.’ This should be followed by 10–14 days of individual or group exercise to ‘raise and maintain an elevated body temperature for at least 1 h each day’… ‘checked by visual assessment of sweating,’ such that a total of 6 weeks dedicated readiness-training has been completed pre-deployment (Ministry of Defence, 2017). In prescribing sub-maximal exercise in time-limited bouts, with allocated rest days (at least 2 per week) and progress toward more thermally-stressful exposures immediately prior to deploying, this approach has the potential to protect, both less and more fit, individuals during preparation for, and on arrival to, a hot environment. In what may be a busy run-in to an overseas exercise or operational mission, unit schedules and individual availability can conceivably limit full compliance with this strategy.

Following arrival to a hot environment, the performance of military duties may usefully be used to induce heat acclimatization, so long as personnel are not critically stressed and/or affected by heat illness in the process. It is common practice (e.g., French and United Kingdom guidance) to promote an initial rest day, with no exercise permitted, in order to allow for transient factors specifically associated with preceding travel that may otherwise undermine heat tolerance (Table 2). The impact of significant troop numbers being incapacitated during early acclimatization can degrade operational capability, challenge the capacity of deployed healthcare systems and, as described above in relation to United Kingdom deployment to Iraq, dictate that many affected personnel are re-patriated (Bricknell, 2003; Bolton et al., 2006). This is particularly the case for severe cases of heat illness or if local healthcare assets lack expertise in the risk stratification and discrimination of casualties whom remain at increased risk of heat stroke, and subsequently require protection from further exposure, or where those with minor heat related debility are removed unnecessarily. The military guidance (Table 2) therefore advocates a slow start to acclimatization, with reduced and time-limited exercise bouts, gradually increasing in intensity and length toward pre-acclimatization norms.

In assessing the impact of progressive aerobic activity added into a program of ‘usual outdoor military activities,’ Charlot et al. (2017) showed an accelerated adaptation versus individuals not exposed to dedicated training, with greater improvements in post-exertional heart rate, thermal discomfort, and RPE following the first week of acclimatization (Charlot et al., 2017). These bouts were of relatively short duration (32 rising to 56 min sub-maximal interval running bouts) by the standards often observed in military protocols, but had the advantage of being complemented by and accommodated within the operationally-focused work schedule mandated from arrival to the hot environment. Performed within military logistic-time constraints the investigators failed to demonstrate a greater reduction in Tc – or blunting of its rise with exertion – in the training group, but cited their improved cardiovascular stability as theoretically conferring better protection against non-heat stroke illnesses, such as heat exhaustion and syncope. Coupled with the significant and substantial reductions in thermal discomfort and RPE observed in both groups following early acclimatization, the authors pointed to the potential for exercise performance to be sustained – and less severe forms of heat illness potentially bypassed – despite limited improvements in heat dissipation and thus Tc responses. The risk of ‘high intensity tasks’ causing adverse effects, such as heat stroke, after 7 days of heat acclimatization, with or without training’ was highlighted. This warning has been echoed in United Kingdom guidance that full acclimatization may take longer than 15 days; that this process may be extended further when a substantial period of travel or crossing of multiple time-zones precedes arrival to the hot environment; and that living or working in air-conditioned accommodation may slow its development (Ministry of Defence, 2017). Charlot et al. (2017) also identified a dissociation between objective physiological variables and subjective psychological variables, with sweat volumes and Tc responses failing to keep pace with large improvements in thermal discomfort and RPE. In a formal evaluation of the United Kingdom regimen provided in Table 1 – undertaken in British paratroopers, marines and specialized infantry, deployed from the United Kingdom to Cyprus, and adapted to incorporate serial testing of heat tolerance (Stacey et al., 2018b) – the most substantial early reductions in exercising heat strain parameters were seen for RPE, followed by heart rate and then Tc (Figure 1). Progress toward final acclimatization status (deployment Day 21) followed the same pattern, with Tc lagging behind RPE and heart rate even after the first full week of the protocol. The need for caution as heat adaptation progresses, highlighted by this relative discrepancy, has been explained as: participants ‘may feel larger improvements in thermal and exercise discomfort, likely increasing intensity during training sessions or operational tasks based on these positive feelings/sensations… (but) the maximum decrease in core temperature was not yet achieved (Charlot et al., 2017). Work conducted in US Navy personnel has shown adaptation discordance between mean skin temperature (>90% progress toward Day 21 acclimatization status after first week) and both heart rate and rectal temperature (only 37 and 38% complete at 7 days, respectively) (Naval Medical Command, 1988) as improved perceptions of the severity of exertion and thermal stress experienced with acclimatization are thought to derive through greater reductions in facial temperature (Malgoyre et al., 2018). The mean lowering of skin temperature may be an important factor in improved physical capacity and the associated potential for sustaining heat stroke – e.g., with unsafe levels of self-paced physical exertion – during and after heat acclimatization.

These data provide a basis to understand how a state of partially complete, or temporarily undermined, heat adaptation may risk more serious forms of heat illness; they would also help to explain the increased hospitalization risk in heat illness cases (Stacey et al., 2015). One factor relevant to this interpretation may be the minimum exposure period recognized as fostering adequate acclimatization. Despite NATO recommendations stating that only a percentage of ultimate adaptation will be achieved with 7 days of protocolized exercise in the heat and United Kingdom guidance highlighting how some individuals will progress more slowly, commanders, and medical personnel alike may fail to appreciate that heat adaptation status has not been adequate to the task required in an individual subsequently affected by heat illness. Based on observations of improved cellular protection and increased organ efficiencies, Horowitz’s group has advanced the case for long term heat acclimatization (LTHA) status taking at least 3 weeks to acquire (Schwimmer et al., 2006). The avoidance of strenuous physical activities until after 3 weeks of adaptation to a new hot environment has long been advocated in military medical practice, at least for deployments where circumstances permit a more graded introduction to heat (World, 2001).

Adaptations to heat exposure is never permanent. According to Givoni and Goldman (1973), heat adaptation is lost for every day spent without heat exposure at a rate that is twice as fast as the rate with which the heat adaptation was initially gained. The lasting effects of heat adaptation, upon removal of heat stimulus, and subsequent re-acclimation has relevance to the military undergoing short notice high intensity missions such as special forces personnel, but also personnel on more enduring operations. The United Kingdom Armed forces, in recent operations, granted its deployed service personnel a leave of rest and recuperation (R&R) for a period of 2 weeks midway through the operational tour. A meta-analysis of 12 studies reported adaptations in end-exercise heart rate decreased by 2.3% for every day of heat acclimation decay. For end-exercise core temperature, the daily decrease was 2.6% (Daanen et al., 2018).

There are several factors, environmental and logistical, which may attenuate or even reverse the physiological effects of heat acclimatization. Whilst some of these factors are specific to the military others are relevant to other occupational groups including firefighters and humanitarian organizations. Core physiological changes in heat adaptation include increased sweat rate, sweating occurring at a lower temperature (Sato et al., 1990) and decreased concentration of sodium and chloride (Conn and Johnston, 1944; Horvath and Shelley, 1946). More dilute sweat is more easily evaporated but only if the climate allows for evaporation (Taylor, 2014; Périard et al., 2015). Certain occupational professionals, particularly firefighters, police and military personnel have requirements for particular uniforms or personal protective equipment due to the nature of the work. These mandatory dress states may attenuate the physiological benefit from sweating adaptation (Smolander et al., 1984; Patton et al., 1995; Maynard et al., 2016) by limiting sweat evaporation even in the context of full acclimatization (Aoyagi et al., 1995). Body armor, having shown to substantially reduce fatal injury to the thorax and abdomen (Mabry et al., 1999; Masini et al., 2009; Belmont et al., 2010) has, over recent conflicts, become commonplace for military personnel, journalists, non-governmental organizations as well as some police units. Studies have indicated that the wearing of body armor increases temperature (Majumdar et al., 1997; Cheuvront et al., 2008; Caldwell et al., 2011) (both core and skin), heart rate (Majumdar et al., 1997; Cheuvront et al., 2008; Caldwell et al., 2011) and produces more sweat (Caldwell et al., 2011) whilst performing military activities (Chinevere et al., 2008; Caldwell et al., 2011). Several studies have outlined an increase in heat stress and thermoregulatory load to chemical warfare suits with a consequential degradation in human performance (Taylor and Orlansky, 1993; Aoyagi et al., 1995). The amount of protection (encapsulation), environmental conditions, physical condition of the soldiers, mission (including duration), amount of physical activity and work rest/cycles appear to be important co-factors (Pandolf et al., 1986; Taylor and Orlansky, 1993). Improved aerobic fitness and the degree of heat acclimation do little to improve the tolerance to CBRN suits during light and moderate exercise (McLellan and Frim, 1994).

Numerous studies have attempted to capture the potential risk factors implicated in heat illness. Commonly cited precipitants include: alcohol (Armstrong et al., 2007), medications (Epstein, 1990; Armstrong et al., 2007) (particularly psychiatric), recreational drugs (Epstein et al., 1999; Armstrong et al., 2007), sickle cell trait (Nelson et al., 2017; Singer et al., 2018), recent febrile illness/diarrheal illness (Epstein, 1990; Armstrong et al., 2007), sleep deprivation (Armstrong et al., 1990, 2007; Bolton et al., 2006; Stacey et al., 2015), sunburn (Armstrong et al., 2007), obesity (Gardner et al., 1996; Epstein et al., 1999; Wallace et al., 2006; Bedno et al., 2014), dehydration (Montain et al., 1998; Armstrong et al., 2007) as well as the known intrinsic factors such as environmental temperature and humidity, clothing and activity levels (Kark et al., 1996; Howe and Boden, 2007). More militarily relevant studies have associated the increased risk of prior heat stroke (Armstrong et al., 2007), combat or healthcare occupational roles, reservists (Bricknell, 1996), subjects over 30 (Epstein, 1990), female sex (Fortney and Senay, 1979; Carter et al., 2005), lower levels of physical fitness (Shvartz et al., 1977b; Epstein, 1990; Gardner et al., 1996; Wallace et al., 2006; Bedno et al., 2014), lack of acclimatization (Epstein, 1990; Bricknell, 1996; Armstrong et al., 2007) and scarred skin areas (Epstein, 1990; Crandall and Davis, 2010; Ganio et al., 2013; Cramer et al., 2019). Caucasian soldiers appear to be at higher risk than African American (Carter et al., 2005). Militaries are drawn from the population of the nations they serve so these risk factors are not generalizable. Many factors can be controlled for using a command structure built on discipline, inherent in military organizations, coupled with careful occupational management. Both systems are fallible however (Cox et al., 2016b). How heat acclimation impacts or mitigates these exacerbating factors is unknown.

Hemorrhage remains the primary modality of battlefield death (Sauaia et al., 1995; Eastridge et al., 2012) commonly before the provision of surgical management (Lind et al., 1968; Crandall et al., 2010b) yet little is known of the effects of heat acclimation on hemorrhagic shock. This is of particular relevance to the military but also civilian aid workers and firefighters. Models, which typically use orthostasis to pool blood in the lower limbs, have demonstrated an inability to tolerate central hypovolemia during simulated hemorrhage when heat-stressed (Lind et al., 1968; Crandall et al., 2010b; Schlader et al., 2016b) although heat acclimation is reportedly protective (Greenleaf and Bosco, 1969; Shvartz and Meyerstein, 1970; Shvartz et al., 1975, 1977a). These findings are not consistent particularly when combining exercise with heat stress in the acclimated (Greenleaf et al., 1974). During heat stress skin blood flow increases significantly requiring a doubling of cardiac output to maintain arterial blood pressure (Schlader et al., 2016b) causing a reduction in central blood volume (Crandall et al., 2012), preload (Wilson et al., 2009), left ventricular end diastolic volume (Nelson et al., 2011) and stroke volume; the proposed mechanisms to how heat stress compromises tolerance to a simulated hemorrhagic insult (Crandall et al., 2019). Whilst heat adaptation, by increasing plasma volume, may improve orthostatic tolerance and reduce heat syncope this may have little effect on actual, rather than simulated, hemorrhage with volume expansion mitigated by dilution of endogenous clotting factors. Furthermore those with a better or worse tolerance to simulated hypovolemia in the heat (Schlader and Crandall, 2014) may not correspond to a better or worse tolerance to hemorrhagic hypovolemia. Heat stress is thought to confer a hyperadrenergic state (Niimi et al., 1997; Cui et al., 2002, 2004, 2010; Low et al., 2011), which potentially reduces the reserve which can be sympathetically activated to maintain blood pressure in the event of a hemorrhagic insult (Crandall et al., 2019). In the military this hyperadrenergic state could be enhanced by sleep deprivation, inadequate nutrition, dehydration (Morgan et al., 2004; Lucas et al., 2013) or mental stress (Greenleaf and Bosco, 1969; Shvartz and Meyerstein, 1970; Shvartz et al., 1975, 1977a, 1981; Yamazaki and Hamasaki, 2003).

Dehydration often used synonymously with hypohydration (Cheuvront and Kenefick, 2014; Kavouras, 2019) and is a common finding in deployed military personnel (Rogers and Cole, 2016; Kavouras, 2019). Heat acclimation potentially guards against dehydration during thermal stress and is associated with reduced markers of renal stress and AKI incidence (Omassoli et al., 2019). Whilst physical conditioning maintains physical work following dehydration, heat acclimatization did not appreciably supplement this effect (Buskirk et al., 2000). Dehydration following heat acclimation may attenuate the effect of heat acclimation in terms of reducing cardiac output during exercise (Montain et al., 1998) and attenuating improvements in cardiac stability. The acclimation derived alterations in core temperature appear to be maintained during dehydration being unaffected by exercise intensity (Montain et al., 1998). Partly due to hypohydration being so ubiquitous in a hot environment, it has been hypothesized that permissive dehydration drives the improved physiological response during heat acclimation (Garrett et al., 2011; Neal et al., 2016) although this remains disputed (Schleh et al., 2018). Permissive dehydration during exercise in the heat may increase the response of fluid regulatory hormones aldosterone, vasopressin, and cortisol (Kenefick et al., 2007) and potentiate acclimation by increased fluid retention resulting in plasma volume expansion (Garrett et al., 2011). Due to concerns regarding heat illness such a strategy would unlikely to gain traction in a military context without further research to address concerns on safety and efficacy.

That acclimatized soldiers succumb to severe heat illness, or heat stroke (Stacey et al., 2015), suggests a dissociation between thermoregulation and heat tolerance (Lim, 2018). There is a growing body of literature suggesting that exertional heat illness is an endotoxemia (Moseley and Gisolfi, 1993; Lim, 2018). A secondary function of the gastrointestinal tract is in the prevention of translocation of potentially harmful luminal antigens into the systemic circulation (Camilleri et al., 2012; Wells et al., 2016). Exercise can adversely disrupt the gastrointestinal barrier integrity (Lambert, 2004) in proportion to the amount of thermal stress (Costa et al., 2017b; Pires et al., 2017) so causing leaking of gram negative bacteria into the intravascular space (Lambert, 2004; Yeh et al., 2013) potentially causing exertional heat stroke (Armstrong et al., 2018; Lim, 2018). Mechanisms include a transient gut hypoxemia secondary to blood shunting from the viscera to exercising muscles causing an overwhelming efflux of endotoxins in circulation (Pyne et al., 2014) and systemic inflammatory responses. Immune stress may further potentiate this with military personnel and aid workers, through sleep deprivation (Day and Grimshaw, 2005; Stacey et al., 2015; Moore et al., 2016), psychological stress (Clow and Hucklebridge, 2001) or repeated (particularly gram negative) infection (Wale, 1989; Grainge and Heber, 2005; Howe and Boden, 2007; Cox et al., 2016a) causing a heightened inflammatory state. Attempts have been made to modulate exercise-induced gastrointestinal permeability with nutritional countermeasures. These include carbohydrates which as well as improving exercise performance (Pöchmüller et al., 2016), immune function (Bermon et al., 2017) and recovery (McCartney et al., 2018) may have a beneficial effect on GI barrier integrity (Gentilcore et al., 2009; David et al., 2014; Edinburgh et al., 2018), although this effect has not been reproducible (Moncada-Jiménez et al., 2009; Sessions et al., 2016; Costa et al., 2017a; Trommelen et al., 2017). Glutamine, an energy substrate of GI enterocytes (Kim and Kim, 2017) is thought to be protective if consumed in higher doses prior to exercise (Zuhl et al., 2014, 2015; Pugh et al., 2017). Bovine Colostrum has been shown to reduce surrogate markers of bacterial translocation and gut permeability following short duration high intensity running (Antonio et al., 2001) but the effects appear attenuated in more demanding exercise protocols in the heat (Morrison et al., 2014; McKenna et al., 2017; March et al., 2019). Whilst multi-strain probiotic supplementation has been shown to reduce endotoxin concentrations in some studies (Shing et al., 2014; Roberts et al., 2016), but not others (Pugh et al., 2019), single strain probiotics may increase concentrations of endotoxin following running in the heat (Gill et al., 2016). Zinc-Carnosine whilst commonly used to treat gastric ulcers (Matsukura and Tanaka, 2000), may also be beneficial in improving gut barrier permiability (Davison et al., 2016). Studies have also assessed the effect of polyphenols such as quercetin during isothermic heat acclimation regimes (Kuennen et al., 2011) or curcumin, a constituent of turmeric (Szymanski et al., 2018), but the effects of these appear negligible (Sergent et al., 2010). Other studied agents include the NO precursors L-arginine (Costa et al., 2014) and L-citrulline (Van Wijck et al., 2014) which also lack objective evidence. Overall there is insufficient reproducibility to recommend the use of large scale nutritional supplementation to prevent heat stroke either during, or as an adjunct, to heat acclimation at present. From a nutrition perspective in terms of heat acclimatization, the military has previously had high salt intakes in their diet (Bannister, 1959) which has the beneficial effect of improving orthostatic tolerance (El-Sayed and Hainsworth, 1996; Cooper and Hainsworth, 2002; Claydon and Hainsworth, 2004). The effect of salt intake on the physiological adaptations following heat acclimation has been studied with the potential of accelerating plasma volume (Armstrong et al., 1993) expansion whilst decreasing heat rate and rectal temperature (Armstrong et al., 1987).

## Optimizing the Risk-Performance Paradigm – the Future of Military Heat Acclimatization

Organizing deployments to allow time for military personnel to acclimate not only improves heat tolerance but can pay dividends in terms of improved physical and cognitive performance (Muza et al., 1988; Chen et al., 1997; Cheuvront et al., 2008; Jovanović et al., 2014). However, mission specific requirements may not allow time for these physiological responses and swifter methods, such as those outlined by Charlot et al. (2017) remain attractive. Whilst, as outlined above, maintaining a high level of physical fitness improves physiological responses to exercise in the heat (Armstrong and Maresh, 1991) by ‘priming’ leading to more rapid acclimation (Pandolf et al., 1977; Garrett et al., 2011) trained individuals may have a lower adaptation response due to higher levels of background adaption secondary to the physical training (Convertino, 1991; Taylor, 2000; Garrett et al., 2011). In contrast whilst slower adaptation is seen in individuals with lower fitness levels the physiological manifestations are reportedly greater (Shvartz et al., 1977b; Garrett et al., 2011). Regardless the untrained, in the military context, appear to be more susceptible to heat illness (Stacey et al., 2016) but whether lower levels of physical fitness confer a risk of heat stroke, with fitter individuals being protected, remains challenging to study due to the numerous confounders.

Commonly an objective assessment of heat tolerance is used by assessing physiological responses to exercise in the heat (Bergeron et al., 2012). In athletes (Karlsen et al., 2015; Racinais et al., 2015) and military personnel (Taylor, 2014) this is commonly performed using a standardized heat tolerance test (HTT). The HTT was developed by the Israeli Defense Force (Epstein, 1990) as a means to occupationally manage their servicemen and women and is commonly used by militaries to differentiate between a temporary and permanent state of heat susceptibility (Moran et al., 2007). The HTT monitors heart rate and core temperature whilst walking for 120 min at 5 km/h at 2% elevation at 40°C and 40% relative humidity. Heat intolerance is confirmed with a core temperature ≥ 38.5°C or a heart rate ≥145 for >3 min (Moran et al., 2007). Whilst recommended by the American College of Sports and Medicine (O’Connor et al., 2013) the HTT, for the military and a multitude of other occupational groups, is a practically and economically impossible way of assessing the presence of heat adaptation. Other studies have attempted to find surrogate markers including early reductions in heart rate and cortisol in the short term followed by diminished excitability by heart rate variability and nephrine measures with respect to LTHA (Stacey et al., 2018b). Elevated copeptin, a surrogate for arginine vasopressin secretion, measured before and after heat stress exposure in military subjects correlated with a core temperature rise greater than 38°C in comparison with subjects where the core temperature rise was less than 38°C (Stacey et al., 2018a). Whilst copeptin may be a surrogate for integrated physiological strain during work in a field environment it is unknown how this is influenced by heat adaptation. The modification of activity in the heat arguably could be better matched to physiological data particularly in the military where optimal performance is not only mission critical but conceivably could mean the difference between life and death. These potentially could include heat acclimatization status which could be combined with fitness, hydration status, core temperature, biomarkers, clothing and equipment and even potentially cumulative heat stress (Hosokawa et al., 2019). With wearable technologies, perhaps in combination with a heart rate target approach (Delves and Buller, 2017; Friedl, 2018), this could prove a safe, effected and targeted method to heat acclimatize military or humanitarian agencies in the future. As with the other occupations and sporting pursuits, a definitive marker of heat adaptation status is one of the Holy Grails of military acclimatization research. As individual responses to heat acclimation are variable (Racinais et al., 2012) reliable markers of adaptation could not only provide assurance but reduce heat illness and lead to more personalized approaches to adaptation (Bergeron et al., 2012; Racinais et al., 2015).

Several novel, often technological, solutions, largely through the reduction of exogenous or endogenous heat stress, have been developed for the purposes of preventing heat illness and potentially improving athletic performance (Walters et al., 2017). Such advances in cooling either improve on the heat adaptive state or bridge the gap prior to full heat acclimation. Skin cooling maintains orthostatic tolerance in heated subjects and reduces heat syncope (Wilson et al., 2002, 2011). As heat loss through sweat evaporation is the only way humans can dissipate heat from the skin these mechanisms commonly aim to enhance or bridge acclimative changes of increased sweat production and evaporation (Candas et al., 1979a, b). The main determinant of evaporation is the vapor pressure gradient between the saturated skin surface and the ambient air which is influenced by skin wettedness, air velocity, and body surface area (Cramer and Jay, 2017). Cooling mechanisms therefore largely exert effects by evaporative cooling, phase change materials, compressed air systems and/or thermoelectric systems (Jovanović et al., 2014). How these systems interact or attenuate a heat adaptive state is unknown. The role of novel cooling systems in protecting personnel from thermal stress would appear more prescient when a rapid robust response is required but there is minimal time to acclimatize, e.g., fighting wildfires or a CBRN scenario. Studies using varying methods often show a reduction in temperature (Cheuvront et al., 2008; Hadid et al., 2008), improved tolerance (Walters et al., 2017) and lower cardiovascular parameters (Muza et al., 1988; Chen et al., 1997). All studies are in small cohorts and none have shown a reduction in heat illness which, along with cost effective considerations, would be required for widespread use. Novel cooling solutions, such as carotid artery cooling (O’Hara et al., 2008), have been studied for the purposes of the acute management of heat stroke but have yet to be studied extensively as a preventative tool. Cooling may be best directed toward the face where studies which attempted to uncouple the psychological from physiological effects of heat acclimatization suggested that the reduction in perceived strain of exertion and thermal stress following acclimatization may be derived through greater reductions in facial temperature (Malgoyre et al., 2018). Whilst there has been significant research in this area, due to the obvious potential advantages, the efficacy of cooling devices, such as cooling vests, are reportedly, and somewhat disappointingly, only as effective as drinking ice cold water (Sawka et al., 1992; O’Hara et al., 2008). In a meta-analysis (Daanen et al., 2018), adaptations in core temperature were more sustained when daily heat exposure duration was increased but heat exposure intensity was decreased. For improvements in sweat rate longer heat acclimation periods are correlated. In contrast core temperature reduction and attenuation appears to be related to higher heat exposure intensity. Importantly, heat re-acclimation appears to induce changes at a faster rate than initial heat acclimation (Daanen et al., 2018). Whether a state of heat acclimation can be maintained for prolonged periods despite repeated loss of heat exposure through rapid heat re-acclimation protocols requires further study in the military context. Potential novel methods include the use of hot baths or saunas (Stanley et al., 2015; Casadio et al., 2017; Mee et al., 2018).

## Conclusion

Heat stress remains a persistent problem for militaries and is likely, with climate change, to represent a challenge in years to come. Militaries are drawn from the populations they serve. The diverse constituents of militaries, coupled with the individual responses to heat acclimation and the varied environments in which militaries operate make generalizations difficult. Thermal stress in modern military training and operational deployments must be reviewed in this context. Heat adaptation remains a mainstay of augmenting physical and mental performance in the military. The ultimate singular goal of a military is to succeed in mission objectives whether in training, contingency operations, or warfighting. The consequences of not understanding what attenuates heat adaptation can pose an existential threat to militaries and their personnel regardless of the threat of heat illness. Heat adaptation in the military should not be reviewed in isolation but in the context of warfighting including wounding, the wearing of personal protective equipment, dehydration and febrile illnesses. Further work should ascertain the mechanisms by which physical fitness and acclimatization may protect against heat illness, over and above the heat syncope mitigated by plasma volume expansion. In the military this is challenging due to the obvious confounder that fitter individuals are more likely to be part of DCCC units and at increased risk of heat illness. Methods of being able to rapidly and safely heat adapt large numbers of varied military personnel, easily measure the adaptive changes and maintain, even manipulate, this adaptation remains a topic of ongoing research. The military remains a special population for thermal research.

## Author Contributions

IP and MS researched, drafted, and edited the manuscript. DW provided editing and overarching review of content.

## Conflict of Interest

The authors declare that the research was conducted in the absence of any commercial or financial relationships that could be construed as a potential conflict of interest. All authors are serving members of the British Army.
